# Neutrophil dynamics in the blood and milk of crossbred cows naturally infected with *Staphylococcus aureus*

**DOI:** 10.14202/vetworld.2015.336-345

**Published:** 2015-03-16

**Authors:** Dilip K. Swain, Mohar Singh Kushwah, Mandheer Kaur, Ajay K. Dang

**Affiliations:** Division of Dairy Cattle Physiology, Lactation and Immunophysiology Laboratory, National Dairy Research Institute, Karnal - 132 001, Haryana, India

**Keywords:** apoptosis, CD44, mastitis, neutrophil, neutrophil extracelluar traps, *Staphylococcus aureus*

## Abstract

**Aim:**

The present study was designed to evaluate the neutrophil dynamics in terms of the functional competence during subclinical mastitis (SCM) and clinical mastitis (CM).

**Materials and Methods:**

A total of 146 Karan fries cows were screened and were divided into three groups as control (n=12), SCM, n=12 and CM, n=12 groups on the basis of California mastitis test scoring, bacteriological evaluation, gross and morphological changes in milk and by counting milk somatic cell count (SCC). Both blood and milk polymorphonuclear neutrophils (PMNs) were isolated in the study. Phagocytic activity (PA) was studied by spectrophotometrically; neutrophil extracelluar traps (NETs) were studied by scanning electron microscopy (SEM); CD44 was quantified by flow cytometry and apoptosis was studied by fluorescent microscopy.

**Results:**

Significantly (p<0.05) higher SCC, PA was found in milk of CM cows as compared to SCM and control cows. Significantly lower (p<0.05) apoptosis was observed in PMNs isolated from both blood and milk of CM group of cows when compared to control and SCM group. The milk neutrophils of CM group of cows formed NETs as evidenced from the SEM images. Surface expression of CD44 revealed a significantly (p<0.05) lower expression in milk neutrophils of CM group of cows when compared to SCM and control group of cows.

**Conclusion:**

The study indicated a positive correlation between delayed neutrophil apoptosis, persistent staying of neutrophils at the site of infection along with formation of NETs as the strategies to fight against the pathogens in the udder during *Staphylococcal* mastitis. The study forms a strong base for future molecular research in terms of neutrophil recruitment and neutrophil removal from the site of infection.

## Introduction

Neutrophils are terminally differentiated cells with a very short life span in the circulating blood and form the first line of cellular defense [1]. Neutrophils form the integral part of mammary innate defense system during foreign intrusion of microbes as an early response of the host body [2]. Multiple factors have been associated with neutrophil trafficking such as the pathogen constituents, cytokines, chemokines and mammary epithelial cells [3] however, many factors are yet to be understood at molecular level [4] pertaining to different pathogen involvement along with their immunogenic components.

The recruitment of neutrophils to the site of pathogen entry has two major aims; firstly neutrophils will employ various mechanisms to kill the pathogens and the second aim is to protect the udder from the next attack of pathogens [5]. Neutrophils after carrying out the process of phagocytosis undergo constitutive apoptosis through cascades of signaling pathways leading to resolution of inflammation [6]. These apoptotic neutrophils are recognized by the resident macrophages, and the macrophages remove them from the site of inflammation to protect the tissue from secondary damage [7]. Macrophages remove apoptotic neutrophils because of expression of CD44 surface receptors on the surface of neutrophils. The receptor expression signals the macrophages to remove the apoptotic neutrophils from the site of infection and thereby prevents the tissue damage by inhibiting the release of neutrophil constituents [8].

*Staphylococcus aureus* is considered as one of the most virulent bacterial species that cause bovine subclinical mastitis (SCM) and clinical mastitis (CM) [9]. The pathogen has been associated with long and persistent forms of infection in cows by employing multiple mechanisms by which it can stay in the neutrophils and prolong the course of inflammation [10]. This involves alteration of the phagocytic activity (PA) and inhibition of complement activation cascades by the prevention of Fc receptor interaction with the antibody immobilized to the surface of the bacteria [11].

In our earlier study in indigenous mastitic Sahiwal (SW) cows, we reported formation of web-like structures by the milk neutrophils [12]. These web-like structures were termed as neutrophil extracelluar traps (NETs). In that study, NETosis was proposed as a mode of cell death during CM. In organized dairy herds of India, the *Staphylococcal* mastitis is considered as the predominant form of SCM and CM. The prevalence rate of this organism counts for more than 50% in India and more than 70% in non-established dairy farms in causing mastitis. Not only this incurs a reduction in production of the cows but also it takes a prolonged course of cure. In Indian prospective, studies are scanty regarding the molecular mechanisms of host-pathogen interaction. Furthermore, pathogen interaction with the host varies depending on the climatic variables and looking to the role played by the pathogen, the present study was proposed to study the neutrophil dynamics both in blood and milk in high yielding Karan Fries (KF) cows. To the best of our knowledge, literature is scanty regarding the functional role and the dynamics of the blood and milk polymorphonuclear neutrophils (PMNs) during the time of *Staphylococcal* mastitis along with there has been no report regarding the formation of NETs in CM in crossbred cows. The relative information regarding the expression of CD44 on the neutrophil surface is also not available in the literature.

Hence, the current study was carried out to study the functional dynamics of both blood and milk neutrophils in control healthy cows and those with subclinical and CM in terms of neutrophil apoptosis, formation of NETs and expression of CD44 surface receptor.

## Materials and Methods

### Ethical approval

Ethical permission for collection of biological samples and their evaluation: Milk and blood samples were collected from the cows as per the guidelines of Institutional Ethical Committee. Prior permission has been taken and approved by the committee for the entire research period.

### Selection of animals for the study

The study was conducted on crossbred KF cows reared under semi-intensive system of management. A total of 146 KF cows were screened during the entire study out of which 36 cows have been selected as per the requirement of the study. Thirty-six cows were taken and categorized into three groups with each group having 12 KF cows. All the cows were screened for mastitis by California mastitis test (CMT), gross/morphological changes in milk, bacteriological culture and microscopic examination of culture and microscopic counting of milk SCC. The determination of CMT score was based on the specifications written on the commercial CMT solution and accordingly the scoring was done (Masti Check Reagent, GEA Westfalia Surge, India). The first group of 12 cows was apparently free of infections; cultured milk samples were negative for the presence of bacteria was classified as control/control group. The second group of 12 cows having CMT score of single positive and milk SCC of 2.48-2.57 (10^5^/ml), and apparently no change in the milk morphology were classified as cows suffering from SCM group. The third group of cows was having CMT score of triple positive based on the intensity of gel formation and SCC count of 6.14-7.86 of 4.5-5.85 (10^5^/ml) were classified as CM group. This group of cow’s milk was showing the presence of flakes and clots with very high milk SCC.

Bacteriological examination of milk samples was carried out as per the method described earlier [12]. Approximately, 0.02 ml aliquot of each sample was spread on 5% sheep blood agar. The plates were incubated aerobically at 37°C and examined after 24 and 48 h. In the case of *S. aureus*, isolates from one colony per inoculums were considered positive. Growth of two different types of colonies per type was considered as a mixed culture. Growth of three or more bacterial types was considered as a contaminated culture and was rejected. The plates having monoculture colonies were further confirmed by morphological assessment and by performing standard biochemical tests.

### Sampling of milk and blood

Milk and blood samples were collected from all the 3 groups of cows as per the protocols approved by the animal ethics committee. Teats were disinfected with 70% of ethyl alcohol prior to collection of milk. A volume of 50 ml of milk was collected in sterile plastic centrifuge tubes. The tubes were collected in the ice box and within 15 min transferred to the laboratory for further processing and evaluation. Autologous blood samples of 9 ml were taken after sampling of milk by venipuncture from the external jugular vein by using heparin as an anticoagulant. Blood and milk samples were collected in the morning between 6.30 am and 7.30 am.

### Isolation of blood neutrophils

All materials and reagents used for the isolation of blood andmilk PMNs were sterile and of cell culture grade (Sigma Chemicals, Germany). Isolation of PMNs from peripheral bloodsamples was performed as per the protocol described [12,13]. Briefly, 10 ml of blood was poured into the falcon tubesand centrifugation was carried out at 1200 × g at room temperature for 20 min for the collection of the hematocrit and this served the source of PMN. 3 ml of the hematocrit were taken in a new falcon tube and was slowly mixed with an equal volume of 1.5% ammonium chloride for lysis. After 5 min, centrifugation at 1000 × g for 10 min was carried out at room temperature for the collection of the cell pellet. The cell pellet was dissolved in 3 ml of phosphate buffer saline (PBS) and the cells were resuspended. Layering of 3 ml cell suspension was carried out slowly over 3ml of Histopaque 1077 (Sigma, Germany) and centrifuged at 1800 × g for 20 min at room temperature for the separation of lymphocytes. The cell pellet formed at the bottom of the falcon tube was considered as PMNs, which was washed 3 times with PBS and centrifuged at 400 × g for 5 min for final suspension in RPMI medium for further analysis and served as the source of blood PMN. The purity of the blood PMN was found to be more than 90% as evaluated by Field’s stain under oil immersion (×100).

### Isolation of milk neutrophils

Isolation of PMN from milk was performed as per the method described [12,13] for milk samples. Briefly, milk was filtered through a nylon filter (40 µm pore size) and diluted to 50% with cold Dulbecco’s PBS (volume/volume). 10 ml of milk was poured into a centrifuge tube and centrifuged (600 × g, 15 min, 4°C) for the removal of fat. The cell pellet was washed twice in cold Dulbecco’s PBS (300 × g, 10 min, 4°C and 200 × g, 15 min, 4°C) and resuspended in Dulbecco’s PBS containing 0.5 mg/ml gelatin. In a 15 ml falcon, 3 ml of Histopaque 1119 (Sigma, Germany) was taken over which 3 ml of Histopaque 1077 (Sigma, Germany) was layered slowly. A ring was formed at the junction of both the Histopaques. Over the Histopaque 1077, 3 ml of cell suspension was layered slowly. The tube was gently placed in the centrifuge, and centrifugation was carried out at 2000 × g at 4°C for 30 min. The cell pellet formed at the interface between the Histopaque 1119 and 1077 was taken as milk PMNs. The collected cells were washed twice with PBS and resuspended in RPMI medium as explained before for further analysis.

### Viability of blood and milk neutrophils

Viability of the blood and milk neutrophils was evaluated by trypan blue (Sigma Chemical Co) staining [12]. We also evaluated viability of neutrophils by employing propidium iodide exclusion by using fluorescent microscopy (IX 51 inverted Olympus microscope).

### Phagocytic activity of blood and milk neutrophils

PA of the blood and milk neutrophils was determined by nitro blue tetrazolium assay (NBT, Sigma Chemical Co) as per the method described by [12,14]. Briefly, the neutrophil suspension was adjusted to 5 × 10^6^ live cells/ml by the culture media (RPMI 1640) containing 10% fetal calf serum. Approximately 200 ml of the diluted cell suspension per well in triplicate was placed in a 96 well flat-bottomed tissue culture plate. The cells were allowed to proliferate with Zymosan (650 µg/ml) and NBT (250 µg/ml) concentrations that had been determined previously to provide maximal stimulation of bovine phagocytes. In all the cases, final culture volume was 200 µl. The blank wells consisted of 200 ml of culture media along with same concentrations of NBT and zymosan. All cultures were allowed to incubate at 37°C in a humidified CO_2_ incubator (95% air and 5% CO_2_) for 2 h. Optical density (OD) was taken at 540 nm multiwall scanning spectrophotometer (Microscan MS-5608A).

### Evaluation of apoptosis in blood and milk PMNs

Apoptosis like changes were evaluated by using two assays.

- Translocation of phosphatidylserine: One of the hallmarks of early apoptosis of cells is characterized by the externalization of phosphatidylserine. This was evaluated by using the Annexin V-FITC apoptosis detection kit as per the protocol described in the kit specifications.
- Evaluation of transmembrane mitochondrial potential of blood and milk neutrophils: The mitochondrial transmembrane potential was measured by employing the MitoPT™ JC-I kit (Immuno Chemistry Technologies, USA, Cat no-924). The method is based on the principle that, the JC-I reagent dye easily penetrates the cells, control and intact mitochondria. Once inside a control non-apoptotic cell, the lipophilic MitoPT-JC-I reagent, bearing a delocalized positive charge, enters the negatively charged mitochondria where it aggregates and fluoresces red. The fluorescent cationic dye, 5,5’,6,6’-tetrachloro-1,1’,3,3’-tetraethyl-benzamidazolocarbocyaniniodide, commonly known as JC-I stains the mitochondria and detects the change in the transmembrane potential of the mitochondria. Cells bearing the control/polarized mitochondria appeared orange-red while cells bearing the depolarized mitochondria appear green.

### Surface expression of CD44 using flow cytometry

Neutrophils isolated from blood and milk was taken at a concentration of 10^5^ cells per ml of the suspension in triplicates. Briefly, 250 µl of cell suspended in PBS were taken in four tubes. The first tube was only having cell suspension without any antibodies and was considered as the control. The second, third and fourth tubes were taken as experimental/test tubes. In each of the test tube, 250 µl of cell suspension was taken and to this the primary antibody was added, which was tagged with FITC as a probe. The primary antibody (CD44-FITC) was used at 1:50 dilutions (Biorbyt, Cat no- orb15300, 0.5 mg/ml). The cell suspension along with the primary conjugated antibody was incubated for 1 h at 37°C in the dark. After 1 hr of incubation, the cell suspension was washed twice with PBS (pH=7.4) at 500 × g for 5 min. After second washing, the cell pellet was resuspended in 250 µl of PBS. To this, 250 µl of 1% paraformaldehyde was added and stored in dark at 4°C till the use. After 24 h of cell labeling, the cells were analyzed for the quantification of expression of CD44.

### Flow cytometry

Mean fluorescence intensity was quantified using a FAC Scan flow cytometer (Becton Dickinson Immunocytometry Systems, BD Bioscience, USA, FACS ARYA). The instrument used the Diva software for the analysis of the data. PMNs were gated on dot plots representing cell size based on forward light scattering and granularity based on side light scattering. 10,000 events were acquired for the evaluation of the relative expression of neutrophil surface receptors in control/control, SCM and CM group samples of blood and milk. During the study, forward light scatter (FSC), side scatter (SSC), FITC fluorescence (FL1) parameters of blood and milk PMNs were quantified by using the flow cytometry. The neutrophils were identified and gated on the basis of their size (FSC) and granularity (SSC). The results obtained were expressed in % expression on neutrophils.

### Scanning electron microscopy of isolated blood and milk neutrophils

Investigation of ultrastructural changes and formation NETs by both blood and milk neutrophils were carried out by employing SEM [12]. Briefly, neutrophils were prepared for SEM in three steps such as fixation, dehydration, and coating. Freshly isolated neutrophils were added to 0.7 ml PBS and 0.3 ml of fixative solution (2.5% Glutaraldehyde, Sigma Chemicals Co). The mixture was thoroughly mixed and centrifuged at 750 × g for 10 min. After centrifugation, the supernatant was removed and to the pellet fresh 0.5 ml of fixative was added. The mixture was kept at 4°C for 2 h. This was followed by removal of fixative by centrifuging at 750 × g for 10 min. The pelleted cells were dehydrated with graded acetone (30%, 50%, 70%, 90% and 100%). Pellet was properly mixed with acetone and suspended in eppendorf tube for 10-20 min. The mixture was gently spinned to obtain a soft pellet, and the supernatant was removed. Finally, the cells were kept in 0.5 ml of 100% acetone and vortexing was carried out. Aluminum paper was kept on stubs and cover slips were kept over it. About 10 µl of Hexamethyl-derilazane, Sigma Chemical Co was added. The sample was diluted with 1.5 ml of 100% acetone and 20 µl of the sample was taken for drying in a laminar hood. This was followed by pallidium coating and carried out for 1.5 h under high pressure and vacuum. After coating, the samples were ready for immediate visualization under microscope. The coated samples were examined under scanning electron microscope (Zeiss Evo maio) at 20KV/EHT and 10Pa between 5000 × and 25000 × after 30 nm palladium coating.

### Statistical analysis

Statistical analysis was carried out by using the Sigma Plot software package version 7.01. Means and standard errors of the mean (S.E.M.) were calculated and the data were presented as means ± S.E.M. To compare the groups with respect to different variables in blood and milk, two-way Analysis of Variance was used and the significance was tested at 0.05 (5%) for all the observations.

## Results

### Milk SCC in cows

**Figure-1:**
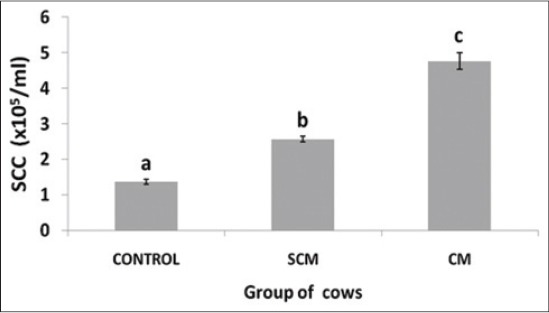
Somatic cell counts (× 10^5^)/ml of milk in different groups of Karan Fries cows. Significance of the difference among the three groups is indicated with asterisks (*p<0.05; n=12). Bars represent the standard error of the mean

In the control group, milk SCC were in the range of 1.11-1.37 (10^5^/ml) of milk, whereas, the SCM group exhibited a range of 2.16-2.89 (10^5^/ml) of milk and the CM groups revealed a highly significant (p<0.05) SCC range between 3.88 and 6.14 (10^5^/ml) in CM group of cows (Figure-1). Control/control animals showed negative CMT score, whereas, the SCM group showed single positive; and the CM group of cows showed double and three positive CM group along with thick gel formation as already discussed in the earlier section.

### Bacteriological evaluation of milk samples

Bacteriological evaluation of milk samples revealed the presence of *S. aureus* in the milk samples of infected cows (both SCM and CM). After screening, we have selected 12 cows each for SCM and CM on the basis of bacteriological examination. The milk samples of control animals were negative for the presence of the pathogen as confirmed from the culture and biochemical tests.

### Percent neutrophil count in blood and milk

**Figure-2:**
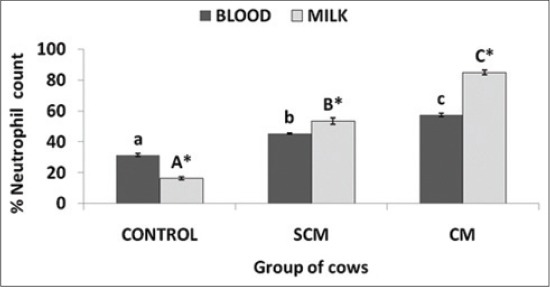
Per cent neutrophil in blood and milk samples in different groups of Karan Fries cows. Significance of the difference among the three groups is indicated with asterisks (p<0.05; n=12). Bars represent the standard error of the mean

Neutrophils isolated from both blood and milk was counted in three different groups of cows. Neutrophil count (%) was significantly (p<0.05) increased in SCM and CM group of cows when compared to the control group. With the severity of infection, the percent count of neutrophils increased as depicted in Figure-2.

### Neutrophil viability and PA of blood and milk neutrophils

**Figure-3:**
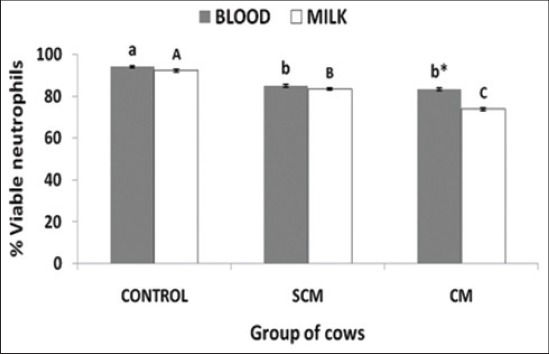
Per cent neutrophil viability in blood and milk of different groups of Karan Fries cows. Significance of the difference among the three groups is indicated with asterisks (p<0.05; n=12). Bars represent the standard error of the mean

The comparative neutrophil viability of both milk and blood has been presented in Figure-3. The control cows showed a significantly (p<0.05) higher viability of both blood and milk neutrophils. The SCM and CM group of cows showed a significantly (p<0.05) lower viability of blood neutrophils when compared to the control cows. The blood neutrophil viability was lower in CM group of cows as compared to the SCM group of cows but was not significant.

**Figure-4:**
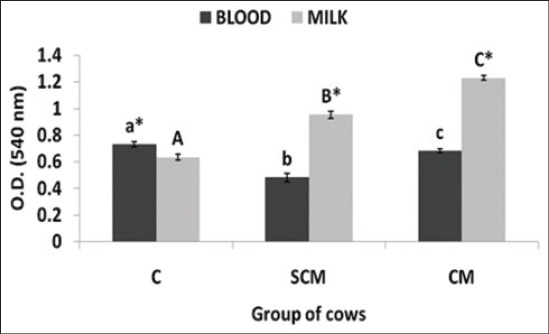
Optical density of the formazan crystals of blood and milk neutrophils of normal, subclinical mastitis and clinical mastitis Karan Fries cows. Significance of the difference among the three groups is indicated with asterisks (p<0.05; n=12). Bars represent the standard error of the mean

PA of the isolated blood and milk neutrophil in terms of OD of the formazan crystals taken at 540 nm has been presented in Figure-4. The PA of milk neutrophils was significantly (p<0.05) higher in both SCM and CM group of cows when compared to the blood neutrophils except in the control group of cows. The PA of milk neutrophils of CM group of cows was significantly (p<0.05) higher with respect to SCM group of cows and PA of SCM group of cows was significantly (p<0.05) higher to the PA of control group. Control group of cows showed a significantly (p<0.05) higher blood neutrophil PA when compared to the SCM and CM group. In control cows, the PA of blood neutrophils was higher when compared to milk neutrophils, but in the SCM and CM group, the milk neutrophils exhibited a significantly (p<0.05) higher PA as compared to blood neutrophils.

### Evaluation of neutrophil apoptosis

**Figure-5:**
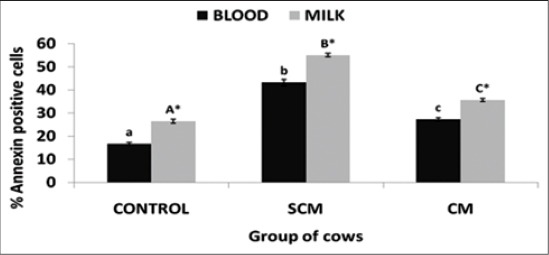
Per cent annexin positive neutrophils of different groups of Karan Fries cows. Significance of the difference among the three groups is indicated with asterisks (p<0.05; n=12). Bars represent the standard error of the mean

Translocation of phosphatidyl serine: Translocation of phosphatidyl serine was evaluated by Annexin V-FITC positive cells in the isolated neutrophils of both blood and milk in different groups of KF cows. The results obtained had been presented in Figure-5. Milk neutrophils exhibited a significantly (p<0.05) higher annexin V positive cells as compared to blood neutrophils in all the three groups of cows. SCM group revealed significantly (p<0.05) higher annexin V positive cells in both blood and milk neutrophils. CM group of cows revealed a significantly (p<0.05) lower apoptosis in both blood and milk neutrophils. The results of CM group were similar to those observed in the control group of cows.

**Figure-6:**
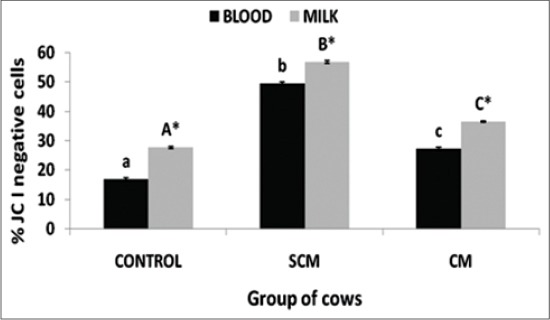
Per cent JC I negative neutrophils of different groups of Karan Fries cows. Significance of the difference among the three groups is indicated with asterisks (p<0.05; n=12). Bars represent the standard error of the mean

Evaluation of mitochondrial transmembrane potential: Mitochondrial transmembrane potential was measured by JC-I staining in KF cows and the results have been depicted in Figure-6. The results revealed a significant (p<0.05) difference in JC-I negative staining among the three groups of cows of both blood as well as milk neutrophils. The rate of JC-I activity significantly (p<0.05) increased in cows with SCM and CM when compared to control. When the results of blood and milk neutrophils were compared, the milk neutrophils exhibited a significantly (p<0.05) higher altered transmembrane potential in all the 3 groups of cows.

Expression of CD44 in blood and milk neutrophils of different groups of cows: CD44 expression was lowest in blood neutrophils of control group and highest in SCM group. CD44 was significantly (p<0.05) higher in SCM group when compared to control and CM group (Figure-7a and b). In all the three groups, the expression of CD44 was significantly (p<0.05) higher in milk neutrophils as compared to the blood neutrophils.

**Figure 7a:**
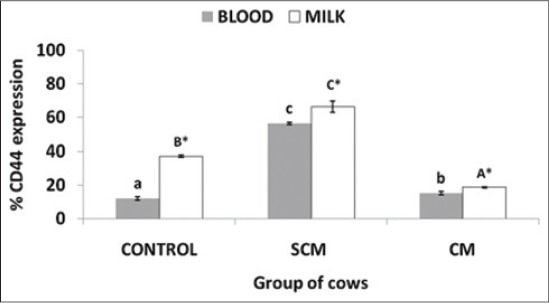
Per cent CD44 expression on neutrophils of different groups of Karan Fries cows. Significance of the difference among the three groups is indicated with asterisks (p<0.05; n=12). Bars represent the standard error of the mean

**Figure 7b:**
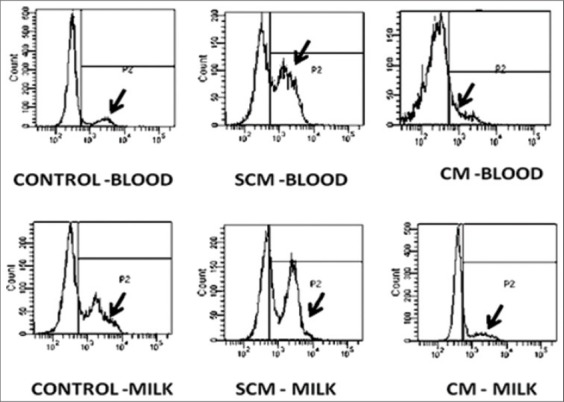
Per cent CD44 expression on neutrophils of different groups of Karan Fries cows. Significance of the difference among the three groups is indicated with asterisks (p<0.05; n=12). Bars represent the standard error of the mean

**Figure 8:**
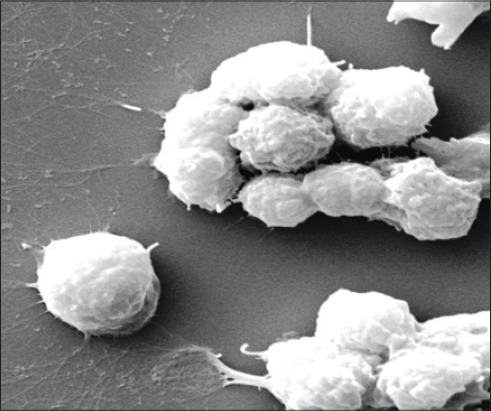
scanning electron microscopy of neutrophils showing formation of neutrophil extracelluar traps (×20000)

**Figure 9:**
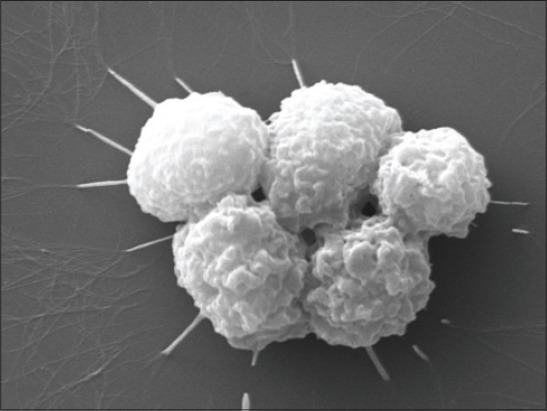
scanning electron microscopy of neutrophils showing formation of neutrophil extracelluar traps (×20000)

Formation of NET: The neutrophils isolated from both blood and milk neutrophils of control/control and SCM group of cows did not reveal the formation of NET like structures. However, the milk neutrophils of mastitic cows revealed the formation of NET like structure. From the imaging, these web-like structures were similar to that of NETs (Figures-8 and 9).

## Discussion

The proposed study was carried out in high yielding KF cows of second to third parity and an average lactational yield of 4500 l of milk. The study was carried out with two major objectives. The first objective was to study the apoptosis in both blood and milk neutrophils isolated from the cows suffering from SCM and CM and the second objective was to study the surface expression of CD44 receptor and the neutrophil dynamics during SCM and CM.

Somatic cells include both the mammary and blood derived cells and considered as an indicator of milk quality and udder health by providing an indication of altered physiology of mammary gland [1]. The predominant cells of SCC are macrophages, neutrophils, lymphocytes and milk-secreting epithelial cells. In the study, SCC significantly (p<0.05) varied among the control, SCM and CM group of cows. SCC increases as a result of inflammatory response of the host to the pathogen and increase in the SCC along with the morphological changes in the milk quality serves as an index for the detection of CM [15]. The insult provided by the pathogen to the mammary gland elicits the host immune response and a resultant compromise in the synthetic activity of mammary epithelial cells [16]. During SCM, there is no change in the gross morphology of the milk but there is an increase in the SCC [17]. In our study, we marked the changes in the milk SCC pattern as reported before by many authors in Staphylococcal mastitis. The level of increase in SCC during SCM is significantly (p<0.05) less when compared to CM. The inflammatory response is predominantly modulated by the pathogen involved, and this may be due to the differential pathogen stimulation to the host. *S. aureus* shows a property of reduced stimulation of the host immune system and also employs various protective tools by which it remains for a very long period in the host immune system without much immune response causing mostly subclinical infections [9].

Viability of the neutrophils is an indication of neutrophil staying in the blood as well as retention in the tissues after their recruitment. The life span of the neutrophils also has been reported to get altered in the presence of pathogens and inflammatory signals [18]. In the study, we reported a reduced viability of both blood and milk neutrophils with the severity of infection. The decrease in the viability of the neutrophils may be due to the pathogen and its associated components in modulating the neutrophil life span [19]. In earlier studies, many authors have reported a delayed neutrophil death during *Staphylococcal and Coliform* mastitis [20-22]. On contrary to this, we reported a lower viability of the neutrophils and this may be due to different experimental design and conditions.

PA is an index to access the neutrophil functional competence. Neutrophils employ phagocytosis as one of the intracellular oxidative method for killing the microbes. The ability to resolve the process of inflammation and protection of the host and host tissues is mediated by the extent and level of neutrophil phagocytosis. The process of phagocytosis is mediated by the degree of activation of neutrophils by the microbes along with the activation surface receptors and downstream signaling in the neutrophils as initiated by the microbes [1,13].

Phagocytosis is also regulated by the localized factors released by the tissues like the cytokines and chemokines. In the study, the SCM and CM group of cows exhibited a significantly (p<0.05) higher PA when compared to the control. As established in the literature, control animals are apparently free of infections and hence the degree of stimulation of neutrophils is less or absent. In the SCM, the moderate degree of stimulation of neutrophils by the microbes occurs as a mode of self defense employed by the neutrophils. In the CM group, the bacteria and bacteria associated components are the potent stimulants of the neutrophil and this may be associated with a higher PA. We also reported higher PA in indigenous SW cows suffering from SCM and CM [12].

Flipping out of phosphatidyl serine is considered as one of the hall marks of early apoptosis. The cells undergoing apoptosis show translocation of phosphatidyl serine and this can be measured by Annexin V-FITC staining technique. We noted a delayed apoptosis of both blood and milk PMNs in clinical mastitic cows. We assume that this may be due to the bacterial cell wall component lipoteichoic acids as reported earlier. In one of our studies in indigenous SW cows, we have reported an up regulation in the toll-like receptor (TLR2) in blood and milk PMNs isolated from cows suffering from CM [12]. Long-term adaptation (LTA) also induces morphological changes in the neutrophils, shedding of selectins (CD62L), degranulation of neutrophils, secretion of cytokines, and activates the oxidative burst mechanisms [9]. We have also reported neutrophil down regulation of CD62L in our earlier studies (data unpublished). Similarly, the LPS as a major component of Gram-negative bacteria, LTA serves as the major component of Gram-positive bacteria. Taking all these information into consideration, it may be possible to infer that the LTA may be the probable factor behind the alterations as well as modulations in neutrophil dynamics during SCM and CM [20].

Phagocytosis induces a non-oxidative mode of cell death in the neutrophils leading to Neutrophil apoptosis [23]. In many studies, it has been found that phagocytosis of microbes by the neutrophils causes accelerated apoptosis in the neutrophils in contrast to the results of our study. In our study, we reported a lower apoptosis of neutrophils. There may be a possibility of involvement of some other signaling pathways, which delay the mode of neutrophil apoptosis during the *S. aureus* mastitis, and this requires further investigation. Yamamoto *et al.*, [23] reported an oxygen-independent pathway involvement in the neutrophils treated with heat-killed *S. aureus* and the mechanism of apoptosis exhibited by the neutrophils is independent to H_2_O_2_.

Bacterial components like LTA acts as a potential activator of neutrophil granulocytes. The component is responsible for the inhibition of apoptosis and enhances the life span of the neutrophils [9]. LTA also activates the neutrophils by activating its degranulation, stimulates the release of pro-inflammatory cytokines, and activates the nuclear factor kappa beta. The interaction of LTA also activates the CD14 receptor and also activates the TLR2 [9,20]. This may be the major factor behind the activation of neutrophils in our study as LTA forms the integral part of the cell membrane of *S. aureus*.

*S. aureus* employs a number of virulence factors like protein A, a capsule or a pseudocapsule (slime), which is anti-phagocytic and a number toxin namely alpha and beta. These factors interfere with the neutrophil functions and also inhibit neutrophil functions, and thereby the pathogen develops clinical or chronic infection [23]. These factors also associated with modulation of neutrophil apoptosis and alteration of apoptosis differentiation program and mediate the bacterial pathogenesis in the host immune system. Studies have shown that, the LTA from the bacteria inhibits the spontaneous apoptosis and increase the life span of the neutrophils. *In vitro* cultivation of blood PMNs also exhibited a delayed DNA fragmentation. In contrast to these, studies have also shown an accelerated apoptosis of neutrophils while they cultivated *in vitro* with the isolates of heat-killed *S. aureus* [20].

Neutrophil transmigration along the vascular endothelium to the tissues under a chemokine-cytokine mediated signaling not only makes the neutrophils to carry out the phagocytosis of the foreign organism, but also provides a greater threat to the tissues. These locomotive neutrophils may cause damage in the migrated tissues. That is why these neutrophils require a process of guided removal from the tissues in a timely manner so as to initiate the process of resolution of inflammation. Neutrophils undergo the constitutive apoptosis in blood due to a short lifespan and degenerated, apoptotic neutrophil cargos are removed by the macrophages [24]. Timely removal of the apoptotic neutrophils is a key requisite for the resolution of inflammation as well as tissue protection and this process is dynamically regulated through complex cascades of signal transduction and cross-talk mechanisms between the neutrophils and macrophages [6].

Phagocytic leukocytes such as neutrophils and macrophages are essential for the innate immune response against invading bacteria. Binding and ingestion of bacteria by these host cells triggers potent anti-microbial activity, including production of reactive oxygen species [23]. Although phagocytes are highly adopt at destroying bacteria, modulation of leukocyte apoptosis or cell death by bacteria has emerged as a mechanism of pathogenesis. Whereas induction of macrophage apoptosis by pathogens may adversely affect the host immune response to infection, acceleration of neutrophil apoptosis following phagocytic interaction with bacteria appears essential for the resolution of infection. This idea is supported by the finding that some bacterial pathogens alter normal phagocytosis-induced neutrophil apoptosis to survive and cause disease [20].

PMNs are typically the first type of leukocyte recruited to sites of infection or areas of inflammation. Ingestion of microorganisms triggers production of reactive oxygen species and fusion of cytoplasmic granules with forming phagosomes, leading to effective killing of ingested microbes. Phagocytosis of bacteria typically accelerates neutrophil apoptosis, which ultimately promotes the resolution of infection. However, some bacterial pathogens alter PMN apoptosis to survive and thereby cause disease [19,24].

Neutrophils undergoing apoptosis are removed from the tissue spaces by the tissue macrophages. This removal of the apoptotic neutrophils is mediated via the surface expression of CD44. Neutrophils undergoing the NET osis may not show the surface expression of CD44 as we did not get any information regarding this during our literature review. In the study, we got a significantly (p<0.05) lower expression of CD44 and assumed that it was due to the lower apoptosis of neutrophils. This finding requires further investigation to reach to a conclusion.

SEM was employed to study the neutrophil surface architecture isolated from blood and milk of control, SCM and CM cows. The SEM images of the neutrophils isolated from milk of CM cows revealed the formation of web-like structures under high magnification. The structures were similar as reported earlier by our group in SW CM milk samples [12]. In our earlier study, we have reported that neutrophils of cows suffering from *S. aureus* infection in the udder show the formation of net like structures known as NETs. Similar pattern of NETs we observed in the present study in milk neutrophils. The blood neutrophils didn’t show the formation of NETs along with the neutrophils isolated from the control/control and SCM cow’s blood and milk. From the study, we did not draw any conclusions regarding the blood neutrophils and formation of NETs. The molecular stimulation for formation of NETs by the neutrophils may be low enough as compared to the milk neutrophils, but this requires further investigation to reach to a conclusion.

Neutrophils employ an extracellular non-oxidative mode of microbe-killing known as NETs by sending the chromatin to the extracellular space [25]. The NETs are rich in proteases that kill the microbes. After the formation of NETs, the neutrophil undergoes a mechanism of cell death independent to apoptosis known as NET osis [26]. This mechanism is poorly understood in CM in dairy cows.

NETs are the extracellular structures, which are formed and derived from the chromatin. These are rich in proteases and are responsible for the entrapment as well as killing of pathogens through a non-oxidative mechanism. The cells after forming the NETs undergo a mechanism of death independent to apoptosis and don’t involve the caspases. This method of cell death is known as NET-osis [25].

In the present study, we observed the neutrophils of both blood and milk isolated from CM cows revealed a significantly (p<0.05) lower apoptosis when compared to the neutrophils isolated from control and SCM cows. We propose another mechanism of cell death known as NET-osis for the neutrophils of CM group of cows. This is a new mechanism employed by the neutrophils to show extracellular killing and do not show any features similar to apoptosis.

## Conclusion

Present study provided an insight into the molecular basis of neutrophil function isolated from crossbred Karan Fries (KF) cows suffering from *Staphylococcal* SCM and CM. From the study, three conclusions were drawn such as, neutrophils show a delayed apoptosis, delayed removal from the tissue spaces and formation of NETs as a mode of innate immune response. Further studies are required to understand the underpinning mechanism behind the process of formation of NETs and how this is associated with neutrophil functions during *S. aureus* infection.

## Authors’ Contributions

DKS and AKD designed the entire study. MSK helped in doing the CMT, SCC and staining of cells. Both DKS and MSK brought the samples from the cattle yard to Lab. MK isolated the cells from blood and milk. DKS carried out all experiments along with analysis of data. AKD finalized the manuscript for communication to the journal. All the authors read the manuscript and approved the final manuscript.
